# Sexual Recidivism During Treatment: Impact on Therapists

**DOI:** 10.1177/10790632231153636

**Published:** 2023-01-19

**Authors:** Michel Raymond, Jean Proulx, Geneviève Ruest, Sébastien Brouillette-Alarie

**Affiliations:** 1 5599 Institut National de Psychiatrie Légale Philippe-Pinel, Montreal, QC, Canada; 2 5622 Université de Montréal, QC, Canada

**Keywords:** recidivism, individuals who have committed sexual offenses, therapists, therapeutic alliance

## Abstract

There are few studies of therapists’ reactions to working with individuals who have committed sexual offenses, and almost none on reactions following sexual recidivism by a patient who is currently in treatment. Consequently, the aim of the current study was to analyze the cognitive and emotional reactions, as well as the intervention strategies, of therapists who have learned of the sexual recidivism of a patient. A total of 59 participants from the province of Quebec (Canada) completed a questionnaire on their reactions to this event. Participants’ responses to their patient’s recidivism varied as a function of gender, experience, and the way they learned of the recidivism. The most common cognitions reported were thinking of the victim and thinking about the consequences of further judicialization for the patient and those close to them. The most common emotions reported were sadness for the victim and fear that the patient would reoffend again. The most common intervention strategies were being sensitive to the experience of the patient and asking the patient what drove them to offend. Support measures for therapists working with individuals who have committed sexual offenses during treatment are discussed.

Treating individuals who have committed sexual offenses may be challenging but also rewarding (Dean & Barnett, 2011). Therapists contribute to a safer society by enabling improvements to clients’ sexual self-regulation skills, socio-emotive skills, and quality of life (Scheela, 2001). There are, however, negative impacts of being involved with persons who have sexually offended.

Farrenkopf (1992) investigated professional burnout, that is, negative effects such as emotional burnout, disconnection, and reduced interest in, and perceived meaning of, work. McCann and Pearlman (1990) and Moulden and Firestone (2010) described vicarious trauma, that is, the process by which the therapist’s perception of themselves, of others, and of the world is negatively affected by their empathy for their patients’ trauma. Edmunds (1997) and Ellerby (1998) introduced the concept of compassion fatigue—distress and emotional depletion resulting from the therapist’s exposure to their patients’ suffering and that of their victims while needing to exhibit empathy for their patients.

One of the main reported repercussions of working with individuals who have committed sexual offenses is a modification of cognitions and emotions, especially tendencies to be more negative, cynical, or discouraged, as well as less trusting of others (Edmunds, 1997; Ellerby, 1997; Farrenkopf, 1992; Rich, 1994). Clinicians also often engage in self-blame for clients’ lack of progress or failings in treatment, assuming that these outcomes are directly linked to their lackluster abilities as a therapist (Dean & Barnett, 2011). Impacts on interpersonal relations have also been reported; these include distrust of strangers and hypervigilance (Edmunds, 1997; Jackson et al., 1997; Steed & Bicknell, 2001), increased feelings of vulnerability, decreased feelings of safety (for either themselves or those close to them; Jackson et al., 1997; Rich, 1994), and isolation (Edmunds, 1997; Farrenkopf, 1992). Therapists may also, in the course of their daily lives, experience intrusive dreams or flashbacks involving thoughts or imagery about sexual violence (Jackson et al., 1997; Rich, 1994; Way et al., 2004).

Hardeberg Bach and Demuth (2018) emphasize the difficulties associated with therapists’ dual responsibility to take into account the interests of both their patients and society. Therapists may therefore find themselves torn between encouraging their patient to open up about their sexual issues and respecting their own obligation to report actual or potential sexual abuses. In addition, since treatment is often court-mandated, some therapists are concerned with the free consent and genuine involvement of convicted persons in these interventions (Chudzik & Ashchieri, 2013; Ward, 2010). Finally, therapists who work with individuals who have committed sexual offenses may not be perceived positively by the community and other professionals (Lea et al., 1999), and may experience hostility from victims and therapists working with victims (Ellerby et al., 1993). Clinician distress has been reported to have impacts on work, especially “emotional hardening” (Scheela, 2001) and difficulties in engaging in effective therapeutic relations (Moulden & Firestone, 2007).

In a study by Farrenkopf (1992), around half of the 24 surveyed therapists seeing individuals with a history of sexual crimes reported reduced hopes and expectations for their patients, increased pessimism about their patients’ potential for change, and increased anger and frustration, which led to the adoption of a more confrontational intervention style. Farrenkopf (1992) described a four-phase process experienced by therapists. The first phase, in the first year of practice with this clientele, is *shock*, due to growing awareness of the prevalence of sexual aggression in society. This phase is associated with feelings of fear, danger, and vulnerability related to their clientele. The second phase, between the first and fifth year, is *mission*, which is marked by therapists' acclimatization to their job and desire to help their patients and make a difference in their lives (and society). In this phase, the therapist is empathic and non-judgmental, and represses negative emotions and reactions related to their patient’s sexual offenses. The third phase is *anger*, in which the emotions repressed in the preceding phase surface, and the therapists adopt an intervention style that is confrontational and intolerant of criminal attitudes. In this phase, the therapist’s idealism is challenged by their patient’s recidivism, defensive attitudes (denial, minimization, rationalization, externalization of responsibility), and cognitive distortions. The fourth phase is *erosion*, and is an extension and amplification of the anger phase. In this phase, therapists are more intolerant, resentful, depressed, and feel that their work is pointless. In Farrenkopf's (1992) study, professional burnout or depressive symptoms were reported by 25% of the participants, and 20% stopped working with individuals who had committed sexual offenses. However, some therapists were able to avoid erosion by increasing their detachment from the outcomes of therapy, as well as their expectations concerning patient change. This adaptation allowed them to regain their motivation, compassion, and continue their work with this clientele.

Even though Farrenkopf’s (1992) model was developed 30 years ago, it is still relevant today, since it concerns therapists’ reactions to difficulties in managing individuals who have committed sexual offenses. Phase 3 (anger) and Phase 4 (erosion) are understandable human reactions by persons involved in the treatment of potentially difficult patients that inevitably challenge the therapeutic alliance. Consequently, coping and supervision strategies must be implemented to manage the negative emotions of therapists, which could, if left unchecked, be detrimental to treatment.

## Factors Influencing the Impact of Working with Individuals Who Have Committed Sexual Offenses

Several studies have reported that the impact of working with individuals who have committed sexual offenses is influenced by several factors, notably the number of years of professional experience with this clientele. While some studies have reported no relation between the number of years and symptoms of vicarious trauma (Way et al., 2004), Steed and Bicknell (2001) observed that therapists with less than 2 years of experience were at higher risk of developing symptoms of vicarious trauma, the same being true for therapists with 9–12 years of experience (due to prolonged exposure; Figley, 1995; Steed & Bicknell, 2001).

The magnitude of the impact of working with individuals who have committed sexual offenses also appears to be a function of workplace characteristics, especially the intensity of exposure (number of patients and contact time) to this clientele (Brady et al., 1999; Chrestman, 1999; Edmunds, 1997; Pearlman & Mac Ian, 1995; Rich, 1994; Woodhouse & Craven-Staines, 2021). Therapists working in secure or correctional facilities, where the level of danger is higher and the atmosphere is more tense, have been reported to exhibit more serious symptoms of vicarious trauma than those working in the community or in outpatient clinics (Ellerby, 1998; Shelby et al., 2001). In addition, distress appears to be higher among therapists who work with incarcerated individuals, as these persons frequently have external motivations for therapy involvement and can be more dangerous, resistant to treatment, impulsive, and manipulative (Moulden & Firestone, 2007).

## Studies of the Impact of the Sexual Recidivism of a Patient on Therapists

Given sexual abuse’s significant consequences for victims and those close to them, the prevention of sexual recidivism is often considered the gold standard of therapeutic success, from the standpoint of both the community and therapists (Jackson et al., 1997). Therapists naturally feel responsible for ensuring the safety of the community, and to this end work on their clients’ rehabilitation and well-being, while carefully evaluating and managing their risk of recidivism. During treatment, difficulties identifying therapeutic progress and doubts about treatment efficacy (particularly with regard to the risk of sexual recidivism) may increase therapists’ stress levels (Farber & Heifetz, 1982).

Encouragingly, meta-analyses have demonstrated that individuals with a history of sexual crime who complete cognitive-behavioral treatment reoffend less frequently than those who do not receive or do not complete treatment (Brouillette-Alarie, 2021; Gannon et al., 2019). For example, Hanson et al. (2009) reported recidivism rates of 10.9% for participants receiving treatment and 19.2% for participants who did not, while Schmucker and Lösel (2015) reported rates of 10.1% and 13.7%, respectively. Although the value of treatment in reducing the risk of recidivism has been demonstrated, some individuals who have committed sexual offenses do nevertheless reoffend.

Currently, studies investigating the impacts of working with individuals who sexually offend are mostly concerned with the general impact of intervening with this clientele, and do not specifically examine the impact of the sexual recidivism of a patient on therapists. Among studies who did, Slater and Lambie (2011) found that when therapists are informed of the recidivism of one of their clients, they may feel betrayed, be concerned about the consequences for the victim, and question their professional competence. Freeman-Longo (1997) points out that the social, emotional, and professional consequences of the sexual recidivism of a patient are probably greater than those associated with other types of therapeutic failure. A parallel can be drawn with therapists who have had a patient commit suicide – a moment described by many as one of the hardest in their careers (Collins, 2003; Hendin et al., 2000).

Ellerby et al.’s (1993) did an exploratory study that evaluated the impact of patients’ sexual recidivism on therapists. Therapists reported that their patients’ recidivism resulted in the following emotions: anger (84%), disillusionment (79%), depression (74%), incompetence (73%), and guilt (42%). Measures taken by therapists to cope with their emotions included venting to colleagues and seeking supervision. The importance of not taking on responsibility for the recidivism, and of analyzing the circumstances and causes of the recidivism, were emphasized. Hardeberg Bach and Demuth (2019), in turn, conducted semi-structured interviews with four therapists in order to evaluate the impact of working with individuals who have committed sexual offenses, and collected data on their reactions to recidivism by patients in treatment. One therapist reported that they felt intensely responsible for a client’s reoffending and wondered about what they could have done differently to prevent it from happening. Another felt angry about the fact that the patient hadn’t used the tools they had acquired in treatment to avoid recidivism.

While relevant, the two studies mentioned above are not without their limitations. Hardeberg Bach and Demuth’s (2019) study is based on only four participants, which severely limits generalizability. In turn, Ellerby et al.’s (1993) study focused on therapists’ emotions and did not examine the cognitions or intervention strategies they adopted when the recidivist was seen again.

## Aim of the Current Study

Considering the lack of scientific literature on the impacts of having a patient sexually reoffend during treatment, or the limitations of existing studies, a new exploratory study was undertaken to analyze the reactions and intervention strategies of therapists who learn of the recidivism of their ongoing patients. Therapists’ cognitive and emotional reactions, as well as intervention strategies upon seeing a reoffending patient, were analyzed in terms of the therapist’s gender, years of experience with this clientele, and the way in which they learned of their patient’s recidivism. Since previous studies were based on small samples and were mainly qualitative in nature (e.g., Hardeberg Bach & Demuth, 2019), we chose to carry out a broader quantitative study based on a larger sample that was representative of the population of therapists involved in the treatment of individuals who have committed sexual offenses in the province of Quebec (Canada). In the following section, we report how we determined our sample size, all data exclusions (if any), all manipulations, and all measures in the study.

## Methods

### Participants

We invited members of the RIMAS (*Regroupement des intervenants en matière d’agression sexuelle;* network of sexual-aggression therapists), an organization located in the province of Quebec (Canada), to participate in a study of their cognitive and emotional reactions, as well as intervention strategies following the sexual recidivism of one of their patients who were in treatment. The study protocol was reviewed and accepted by the ethics and scientific committees of the *Institut national de psychiatrie légale Philippe-Pinel*, a maximum-security psychiatric institution.

RIMAS is a network of approximately 130 Quebec therapists working in various settings (correctional facilities, hospitals, community organizations, private practice) with individuals who have committed sexual offenses. Most of the therapists in this network see patients as part of community treatment. Although not all Quebec therapists working with individuals who have committed sexual offenses are members, the network does represent a high percentage of these professionals. However, it should be noted that some professionals, notably psychiatrists, are underrepresented in RIMAS.

Every member of RIMAS, including those who had never had a patient reoffend during treatment, was invited to complete the French questionnaire. On around 130 potential respondents, 59 therapists accepted to participate in our study. Twenty-nine completed the online version, while 30 completed a paper version at the beginning of a workshop at the June 2019 RIMAS conference. Eighty-five percent of the respondents who chose to participate in the study (50 out of 59) had a patient sexually reoffend during treatment.

### Instruments

The questionnaire (available in the Supplementary Materials, translated into English) was developed by the lead researchers, who have substantial clinical experience in working with individuals who sexually offend. It comprised 26 questions on the cognitive/emotional reactions and intervention strategies of therapists following the sexual recidivism of one of their patients in treatment. The first 11 questions covered age, gender, profession, work environment, therapeutic approach, years of experience with individuals who have committed sexual offenses, and percent of clientele who have committed sexual offenses. Subsequent questions covered the cognitive and emotional reactions, as well as the intervention strategies adopted, following the case of sexual recidivism by a patient that affected the respondent the most. Respondents were asked to specify whether they had learned of the recidivism from the patient or a third party (police, parole officer, probation officer, therapist, someone close to the patient, media). The emotional reactions listed as possible responses were largely inspired by the emotional reactions reported by participants in Ellerby et al.’s (1993) study. The cognitive reactions and intervention strategies listed as possible responses were inspired by statements frequently reported by therapists who have had a patient reoffend. One question inquired about changes in professional practice triggered by the recidivism. Because it was impossible to predict every possible response, respondents were given the opportunity, through an open-ended question, to discuss issues that had not been covered by the other questions. The responses to this last question constituted the qualitative part of our study.

In this study, sexual recidivism was defined as: (1) a contact or non-contact (voyeurism, exhibitionism, telephone harassment, online luring, consumption of child pornography) sexual offense that resulted in an arrest; or (2) a contact or non-contact sexual offense divulged by a patient. To be eligible, the offense had to have occurred while the patient was receiving treatment—that is, between two appointments, regardless of the lapse of time between them. This excluded recidivism which occurred after treatment had terminated. This exclusion criterion was applied because one of our main objectives was to review intervention strategies adopted upon seeing the patient after the recidivism.

### Analytical Strategy

Analysis proceeded in two steps. In the first step, the sociodemographic characteristics of participants were established, followed by an analysis of participants’ cognitive and emotional reactions when they learned that their patient reoffended, and of their intervention strategies upon subsequently seeing the patient. These reactions and strategies were analyzed for the sample as a whole, and were also broken down by the following dichotomous variables: (1) gender (male, female); (2) number of years of experience (fewer than/at least 10 years) with individuals who have committed sexual offenses; and (3) source of information concerning the recidivism (patient or third party). Phi coefficients were used to estimate effect size and determine whether intergroup differences were statistically significant.

In the second step, conceptual scales of the main types of cognitions, emotions, and intervention strategies were created by summing thematically related dichotomous variables. Cognitions were classified as internal or external; emotions were classified as anger or anxiety/disappointment/discouragement; and strategies were classified as empathy-oriented or accountability-oriented. Some cognitions, emotions, and intervention strategies did not conceptually fit into any of the scales, and were not further analyzed.

The “cognitions (external attribution)” score was equal to the sum of the responses to: (a) I’m not surprised, since sexual aggressors are at high risk of recidivism; (b) the patient betrayed me; (c) the patient wasn’t honest with me; and (d) these patients are untreatable. The “cognitions (internal attribution)” score was equal to the sum of the responses to: (a) I failed—am I effective in my work?; (b) I thought I understood the patient, but I was wrong; (c) I should have seen it coming; (d) I misjudged our therapeutic alliance; and (e) I should have done something differently. The “emotions (anger)” score was equal to the sum of the responses to: (a) frustration/anger and (b) disappointment with the patient. The “emotions (anxiety/disappointment/discouragement)” score was equal to the sum of the responses to: (a) guilt; (b) disappointment with yourself; (c) fear of being held responsible, of having to defend your work; (d) discouragement/depression; (e) fear that the patient offend yet again; (f) sadness for the patient; (g) sadness for the victim; and (h) disillusionment. The “intervention strategies (empathy-oriented)” score was equal to the sum of the responses to: (a) sensitizing the patient to the harm caused to the victim; (b) sensitizing the patient to the harm caused to those close to them; (c) ensuring that the patient is aware of their progress, despite the recidivism; and (d) exhibiting sensitivity to the experience of the patient. Finally, the “intervention strategies (accountability-oriented)” score was equal to the sum of the responses to: (a) asking the patient why they had reoffended; and (b) asking why the patient lacked openness and failed to take advantage of the therapeutic work.

Descriptive analysis of these subscales, as well as non-parametric correlation analyses (Spearman) between them, were conducted. Analyses were performed on the whole sample only, as any subgroups would have been so small as to raise issues of representativeness. Nonparametric correlations were preferred over Pearson correlations, given the non-normal distribution of the conceptual scales and the relatively small sample size. Since we have a small sample, effect sizes that were moderate (*r* = .243, according to Rice & Harris, 2005) but non-statistically significant at .05 were still given consideration.

## Results

### Sociodemographic and Occupational Profiles of Therapists

The sociodemographic and occupational characteristics of the therapists who participated in this study are listed in Table 1. The mean age of participants was 41 years, the majority of participants were female, and the most common therapeutic approach was cognitive-behavioral.

**Table 1.** Descriptive Statistics (*N* = 59).

	Mean (SD)/%	Min	Max
Age	41.3 (11.9)	22	65
Gender	74.6% female	—	—
	25.4% male		
Occupation			
Sexologist	34.5%	—	—
Psychologist	22.4%	—	—
Criminologist	15.5%	—	—
Psychotherapist	10.3%	—	—
Social worker	5.2%	—	—
Psychiatrist	3.4%	—	—
Correctional agent	3.4%	—	—
Psychoeducator	3.4%	—	—
Nurse	1.7%	—	—
Work environment^a^			
Community organization	55.9%	—	—
Hospital	27.1%	—	—
Private practice	23.7%	—	—
Correctional facilities	8.5%	—	—
Youth center	5.1%	—	—
Therapeutic approach^a^			
Cognitive-behavioral	89.8%	—	—
Humanist/existential	25.4%	—	—
Psychoanalytic/psychodynamic	18.6%	—	—
Familial/systemic	15.3%	—	—
Pharmacological	1.7%	—	—
Years of experience treating individuals who have committed sexual offenses	12.6 (10.3)	0.0	33.0
Years of experience working in a team	12.0 (8.9)	0.0	32.0
Years of experience working alone	4.6 (9.0)	0.0	30.0
Percentage of therapists’ clientele who are individuals with a history of sexual crimes	76.6 (27.8)	10	100
Percentage legally required to attend	78.8 (26.0)	0	100
Percentage attending voluntarily	20.5 (24.9)	0	100
Percentage of clientele who are victims of sexual assault	8.2 (15.1)	0	70

^a^Total exceeds 100% because some participants reported more than one workplace/therapeutic approach.

A variety of occupations were represented in the sample; the most common were sexologist, psychologist, criminologist, and psychotherapist. Similarly, a variety of work environments—especially community organizations, hospitals, and private practice—were represented. On average, participants had 13 years of experience in the treatment of individuals who have committed sexual offenses, with the median being 10 years. A strong majority of participants had a clientele composed solely of adult males. Some participants worked with adolescent males, but virtually none worked with adolescent or adult females. Most of the patients were legally required to attend treatment. Very few participants saw both perpetrators and victims.

### Cognitions, Emotions, and Intervention Strategies Upon Learning of the Sexual Recidivism of a Patient

Of the 59 questionnaire respondents, 50 had had at least one patient sexually reoffend during treatment. The following analyses are based on these 50 alone. Recidivism took the form of sexual touching or invitation to sexual touching (40%), non-contact sexual offenses in the presence of the victim (26%), offenses involving child pornography/online luring/harassing phone calls (22%), aggravated sexual assault (10%), and other sexual offenses (2%). Of the recidivists, 79.6% had victimized minors and 28.6% had victimized adults (these figures imply that some patients had victimized both types of victims). Participants learned of the recidivism from a third party in 66% of cases. In the remaining 34% of cases, participants learned of the recidivism from the patient, either before legal procedures had commenced or, in some cases, even when no such procedures were ever instituted.

The cognitions participants reported upon learning of the sexual recidivism of a patient are listed in Table 2. The cognitions reported by at least one third of participants are, in descending frequency: (1) thinking about the victims; (2) thinking about the consequences for the patient and those close to them; (3) thinking that the patient had not been honest with them; (4) regretting not having seen the recidivism coming; (5) realizing that they had not completely understood the patient; (6) realizing they had seen the warning signs; and (7) realizing they had misjudged the strength of the therapeutic alliance.

**Table 2.** Cognitions Upon Learning of the Sexual Recidivism of a Patient (*N* = 50).

	Total sample, %	F, %	M, %	Phi	<10 y experience, %	≥10 y experience, %	Phi	Third-party announcement, %	Patient announcement, %	Phi
These patients are untreatable	4.0	5.6	0.0	.13	4.8	3.4	.03	6.1	0.0	.15
I’m not surprised, since sexual aggressors are at high risk of recidivism	10.0	**2.8**	**28.6**	.39**	4.8	13.8	.15	**15.2**	**0.0**	**.24**
I failed—am I effective in my work?	28.0	**36.1**	**7.1**	.29*	38.1	20.7	.19	27.3	29.4	.02
I should have seen it coming	42.0	**50.0**	**21.4**	**.26**	**57.1**	**31.0**	**.26**	39.4	47.1	.07
I should have done something differently	26.0	30.6	14.3	.17	**42.9**	**13.8**	.33*	21.2	35.3	.15
I thought about the victims	60.0	61.1	57.1	.04	66.7	55.2	.12	63.6	52.9	.10
I thought about the consequences for the patient and those close to them	58.0	55.6	64.3	.08	66.7	51.7	.15	54.5	64.7	.10
The patient wasn’t honest with me	56.0	55.6	57.1	.01	52.4	58.6	.06	**66.7**	**35.3**	.30*
I thought I understood the patient, but I was wrong	38.0	36.1	42.9	.06	33.3	41.4	.08	39.4	35.3	.04
I misjudged our therapeutic alliance	34.0	33.3	35.7	.02	33.3	34.5	.01	**42.4**	**17.6**	**.25**
The patient betrayed me	8.0	8.3	7.1	.02	4.8	10.3	.10	12.1	0.0	.21
I saw the warning signs—I’m not surprised	36.0	38.9	28.6	.10	42.9	31.0	.12	30.3	47.1	.17
I must report the patient to legal or youth-protection authorities	26.0	**36.1**	**0.0**	.37**	**38.1**	**17.2**	**.24**	21.2	35.3	.15
I must maintain the patient’s confidentiality, and continue our therapeutic work	18.0	16.7	21.4	.06	19.0	17.2	.02	**6.1**	**41.2**	.43**

*Notes*. F = female, M = male; *p < .05; **p < .01. Statistically significant differences and/or moderate effect sizes are indicated in bold. Analyses are based on the 50 participants who had one of their patients sexually reoffend during treatment.

Female participants were more likely than men to want to report their patient to legal authorities, to question their efficacy as a therapist, and to believe they should have seen the recidivism coming. Male participants, on the other hand, were more likely to find the sexual recidivism unsurprising, given their impression that individuals who sexually offend are at high risk of recidivism. Therapists with less experience were more likely than more experienced ones to believe that they should have seen the recidivism coming, should have done something differently, and wanted to report the patient to authorities in higher proportion. Finally, participants who learned of the recidivism from a third party, rather than from the patient, were more likely to believe that the patient had not been honest with them, that they had misjudged the strength of the therapeutic alliance, and that the recidivism was unsurprising given their perception that this clientele is at high risk of recidivism. On the other hand, participants who learned of the recidivism from the patient were more likely to want to protect the patient’s confidentiality and continue their therapeutic work.

The emotions the participants reported having had upon learning of the sexual recidivism of a patient are listed in Table 3. The emotions reported by at least one third of participants were, in decreasing frequency: (1) sadness for the victims; (2) disappointment with the patient; (3) frustration/anger; (4) fear that the patient would offend yet again; (5) sadness for the patient; (6) disappointment with themselves; and (7) surprise/incomprehension.

**Table 3.** Emotions Upon Learning of the Sexual Recidivism of a Patient (*N* = 50).

	Total sample, %	F, %	M, %	Phi	<10 y experience, %	≥10 y experience, %	Phi	Third-party announcement, %	Patient announcement, %	Phi
Surprise/incomprehension	36.0	38.9	28.6	.10	47.6	27.6	.21	42.4	23.5	.19
Frustration/anger	44.0	44.4	42.9	.01	33.3	51.7	.18	51.5	29.4	.21
Sadness for the patient	42.0	38.9	50.0	.10	**61.9**	**27.6**	.34*	**30.3**	**64.7**	.33*
Sadness for the victims	64.0	58.3	78.6	.19	71.4	58.6	.13	69.7	52.9	.17
Disappointment with the patient	52.0	**44.4**	**71.4**	**.24**	57.1	48.3	.09	**63.6**	**29.4**	.33*
Disappointment with yourself	38.0	41.7	28.6	.12	38.1	37.9	.00	39.4	35.3	.04
Discouragement/depression	26.0	**33.3**	**7.1**	**.27**	28.6	24.1	.05	30.3	17.6	.14
Disillusionment	22.0	25.0	14.3	.12	**42.9**	**6.9**	.43*	27.3	11.8	.18
Guilt	28.0	**36.1**	**7.1**	.29*	23.8	31.0	.08	24.2	35.3	.18
Fear of being held responsible, of having to defend your work	18.0	19.4	14.3	.06	9.5	24.1	.19	21.2	11.8	.12
Fear that the patient offend yet again	44.0	50.0	28.6	.19	**61.9**	**31.0**	.31*	42.4	47.1	.04

*Notes*. F = female, M = male; *p < .05; **p < .01. Statistically significant differences and/or moderate effect sizes are indicated in bold. Analyses are based on the 50 clinicians who had one of their patients sexually reoffend during treatment.

Female participants were more likely than male ones to feel guilty, discouraged, or disappointed. Male participants, on the other hand, were more likely to be disappointed with the patient. Less experienced participants were more likely to be disillusioned, sad for the patient, and fear that the patient would offend yet again. Finally, participants who learned of the recidivism from the patient were more likely to feel sadness for the patient, while those who learned of it from a third party were more likely to be disappointed with the patient.

The intervention strategies adopted by participants upon seeing the reoffending patients are listed in Table 4. As 15 of the participants were no longer in contact with the patient when they learned of the recidivism, they were unable to adopt any intervention strategy, and were therefore excluded from the analysis.

**Table 4.** Intervention Strategies Upon Learning of the Sexual Recidivism of a Patient (*N* = 35).

	Total sample	F	M	Phi	<10 y experience, %	≥10 y experience, %	Phi	Third-party announcement, %	Patient announcement, %	Phi
Asking the patient why they had reoffended	74.3%	68.0%	90.0%	.23	72.7	75.0	.02	78.3	66.7	.19
Asking the patient why they hadn’t been open about the early warning signs they had experienced	68.6%	68.0%	70.0%	.02	**45.5**	**79.2**	.34*	**82.6**	**41.7**	**.33**
Confronting the patient about their lack of openness and failure to take advantage of the therapeutic work	25.7%	20.0%	40.0%	.21	**0.0**	**37.5**	.40*	34.8	8.3	.21
Exhibiting sensitivity to the experience of the patient	80.0%	76.0%	90.0%	.16	**100.0**	**70.8**	.34*	73.9	91.7	.12
Sensitizing the patient to the harm caused to the victim	40.0%	36.0%	50.0%	.13	54.5	33.3	.20	39.1	41.7	.07
Sensitizing the patient to the harm caused to those close to them	37.1%	32.0%	50.0%	.17	**54.5**	**29.2**	**.24**	39.1	33.3	.15
Helping the patient realize the consequences of their behavior on themselves	65.7%	68.0%	60.0%	.08	**100.0**	**50.0**	.49**	60.9	75.0	.03
Ensuring that the patient is aware of their progress, despite the recidivism	**40.0**	**48.0**	**20.0**	**.26**	**63.6**	**29.2**	**.33**	34.8	50.0	.17

*Notes*. F = female, M = male; *p < .05; **p < .01. Statistically significant differences and/or moderate effect sizes are indicated in bold. Analyses are based on the 35 clinicians who had one of their patients sexually reoffend during treatment and saw them thereafter.

The intervention strategies adopted by at least one third of participants upon seeing their patient after the recidivism were, in decreasing frequency: (1) exhibiting sensitivity to the experience of the patient; (2) asking the patient why they had reoffended; (3) asking the patient why they hadn’t been open about the early warning signs they had experienced; (4) helping the patient realize the consequences of their behavior on themselves; (5) sensitizing the patient to the harm caused to the victim; (6) ensuring that the patient is aware of their progress, despite the recidivism; and (7) sensitizing the patient to the harm caused to those close to them. Women were more likely than men to ensure their patient was aware of the therapeutic progress made despite the recidivism. Experience exerted an influence in multiple ways. Less experienced participants were more likely to adopt empathy-based approaches, such as attempting to help the patient realize the consequences of their behavior on themselves and others close to them, being sensitive to the experience of the patient, and attempting to ensure that the patient was aware of their progress despite the recidivism. More experienced therapists were more likely to have intervened in an accountability-oriented manner, focusing on their patient’s lack of openness, especially about early warning signs, and failure to take advantage of therapy. Finally, participants who learned of the recidivism from a third party were more likely to ask the patient about their lack of openness about early warning signs.

### Correlations Between Cognitions, Emotions, and Intervention Strategies

To identify potential links between the participants’ cognitions, emotions, and intervention strategies, six conceptual variables, based on the sum of variables that were thematically similar, were created: cognitions (external attribution of responsibility), cognitions (internal attribution of responsibility), emotions (anger), emotions (anxiety/disappointment/discouragement), intervention strategies (empathy-oriented), and intervention strategies (accountability-oriented).

There was no difference in the mean scores of male and female participants on any of the scales. However, more-experienced professionals experienced less anxiety/disappointment/discouragement (*M* = 2.4, *SD* = 1.8 vs. *M* = 3.4, *SD* = 2,0; *t* = 1.79, *p* < .10) and adopted less empathy-oriented intervention strategies (*M* = 2.1, *SD* = 1.5 vs. *M* = 3.7, *SD* = 1.0; *t* = 3.30, *p* < .01) than less-experienced professionals. Participants who learned of the recidivism from a third party rather than from their patient attributed more responsibility for the recidivism to external factors (*M* = 1.0, *SD* = 0.8 vs. *M* = 0.4, *SD* = 0.5; *t* = 2.95, *p* < .05) and felt more anger (*M* = 1.2, *SD* = 0.8 vs. *M* = 0.6, *SD* = 0.6; *t* = 2.65, *p* < .05).

The Spearman correlation coefficients between types of cognitions, emotions, and intervention strategies are presented in Table 5. Participants with high scores on the “cognitions (external attribution)” scale experienced more anger than anxiety/disappointment/discouragement, while participants with high scores on the “cognitions (internal attribution)” scale experienced both anger and anxiety/disappointment/discouragement, although the correlation with anxiety/disappointment/discouragement was stronger. Unsurprisingly, anger was associated with accountability-rather than empathy-oriented interventions. In turn, participants experiencing anxiety/disappointment/discouragement were more likely to adopt empathy-oriented strategies than accountability-oriented ones.

**Table 5.** Correlation Between Cognitions, Emotions, and Intervention Strategies.

	Cognitions (external attribution)	Cognitions (internal attribution)	Emotions (anger)	Emotions (anxiety/disappointment/discouragement)	Empathy-oriented interventions	Accountability-oriented interventions
Cognitions (external attribution)	—	—	—	—	—	—
Cognitions (internal attribution)	*r* = .36* *n* = 50	—	—	—	—	—
Emotions (anger)	*r* = .64*** *n* = 50	*r* = .43** *n* = 50	—	—	—	—
Emotions (anxiety/disappointment/discouragement)	*r* = .05 *n* = 50	*r* = .54*** *n* = 50	***r* = .24 ***n* = 50	—	—	—
Empathy-oriented interventions	*r* = −.04 *n* = 35	*r* = −.02 *n* = 35	*r* = .13 *n* = 35	***r* = .32 ***n* = 35	—	—
Accountability-oriented interventions	*r* = .14 *n* = 35	*r* = .03 *n* = 35	***r* = .27** *n* = 35	*r* = −.01 *n* = 35	*r* = .06 *n* = 35	—

*Notes*. *p < .05; **p < .01; ***p < .001. Statistically significant differences and/or moderate effect sizes are indicated in bold.

### Supplementary Analyses

Supplemental analyses were conducted to determine whether the cognitive/emotional reactions and intervention strategies of therapists working with both perpetrators and victims of sexual offenses differed from those working solely with perpetrators. The only significant result was that participants who worked with both perpetrators and victims tended to experience more anxiety/disappointment/discouragement upon learning of the sexual recidivism than did those working solely with perpetrators (*M* = 3.9, *SD* = 2.3 vs. *M* = 2.2, *SD* = 1.4; *t* = 3.36, *p* < .01).

Changes in practice following the news of a patient’s recidivism were analyzed using a total-score scale of modified practices. The results indicate that there was a positive correlation between the number of changes in practice and: (a) attribution of the recidivism to internal factors (*r* = .30, *N* = 50); (b) feelings of anger (*r* = .28, *N* = 50); (c) feelings of anxiety/disappointment/discouragement (*r* = .29, *N* = 50); and d) adoption of empathy-oriented intervention strategies (*r* = .41, *N* = 35). Unsurprisingly, external attribution was not a strong driver of introspection and, by extension, of changes in practice (*r* = .07, *N* = 50). Gender, years of experience, and learning of the recidivism from the patient had no impact on the number of changes in practice.

## Discussion

To our knowledge, no empirical study has specifically investigated the impact the sexual recidivism of a patient has on therapists. In addition, this study appears to be the first to have analyzed therapists’ cognitive reactions and intervention strategies adopted upon seeing the patient after recidivism happened.

### Cognitive Reactions

Our results with regard to cognitive reactions indicate that the first reactions of most participants upon learning of the sexual recidivism of their patient was to think of the victims, and of the consequences of further judicialization for the patient and those close to them. Female participants were more likely than their male counterparts to want to report the patient to legal authorities, to question their efficacy as therapists, and to think that they should have seen the recidivism coming. Valente and Sanders (2002) observed a high prevalence of these types of reactions among nurses and therapists who have had a patient commit suicide. In the current study, male participants were more likely to think that the recidivism was unsurprising, given the high risk they perceive individuals who have committed sexual offenses pose. This more detached attitude appears to have been a way to reduce the intensity of their emotional reactions. Participants with fewer than 10 years of experience were more likely to question their efficacy and believe that they should have seen the recidivism coming.

Sexual recidivism appears to have had repercussions on the personal life of the participants. One participant reported that “the recidivism of one of my patients led me to be more vigilant with my children and to inform them of the risks.” Another participant reported having “regularly gone over all our meetings.” This last type of reaction, as well as self-examination regarding warning signs that could have been ignored or underestimated, has also been reported by therapists having had a patient commit suicide (Valente & Sanders, 2002).

The way participants learned of the recidivism influenced their perception of therapeutic alliance. Participants who learned of the recidivism from a third party were more likely to believe that the patient had not been honest with them and that they had misjudged the strength of the therapeutic alliance. Those who learned of the recidivism from the patient felt the patient had trusted them enough to confide in them, and were more motivated to maintain the patient’s confidentiality and continue therapy. It should be noted that not all offenses were of a nature that required reporting to authorities.

### Emotional Reactions

Participants’ reported emotional responses following the sexual recidivism of a patient were more varied than those reported by Ellerby et al. (1993). In this study, participants reported sadness for the victim, fear that the patient would offend yet again, and sadness for the patient. The qualitative responses also revealed feelings of shame, incompetence, and powerlessness. Feelings of anger, disillusionment, and disappointment were less frequently reported than in Ellerby et al.’s (1993) study. Participants who saw both victims and perpetrators of sexual offenses experienced more feelings of anxiety/disappointment/discouragement following the sexual recidivism, which may be explained by their exposure to the experience and suffering of victims of sexual offenses.

Therapists who have had a patient commit suicide are also reported to experience sadness and guilt, as well as shock, surprise, and incredulity (Hendin et al., 2000). In the current study, 36% of participants reported having experienced surprise and incomprehension following sexual recidivism. While therapists who have had a patient commit suicide may frequently feel guilty and fear being either legally prosecuted or blamed by their colleagues, employer, or the patient’s family (Hendin et al., 2000), these fears were only reported by 18% of the participants in this study. This discrepancy may be due to the extensive experience of the participants or to the fact that prosecution of therapists working with individuals who have committed sexual offenses are uncommon in Quebec.

The emotions reported following recidivism varied as a function of gender, years of experience, and source of information regarding the recidivism. Female participants reported feeling more discouraged, disappointed, guilty, and were more likely to question their efficacy. Male participants, in contrast, were more likely to be disappointed in the patient. Female participants appeared to be more likely to take on some responsibility for the sexual recidivism. One female participant reported “feeling like I had not done enough for the patient.” Feelings of guilt, shame, and personal doubt have been reported among female participants who have had a patient commit suicide (Grad et al., 1997). Next, participants with fewer than 10 years of experience were more likely to be afraid that the patient would reoffend again. This parallels the results of Farrenkopf (1992), who observed that therapists who were in the early stages of their career were more strongly affected by the perceived high prevalence of sexual aggression in society. In the current study, participants with fewer than 10 years of experience were also more likely to report feelings of disillusionment. Recidivism thus appears to have contributed to erosion, again paralleling Farrenkopf’s (1992) results. In that study, therapists with more than 10 years of experience experienced less disillusionment, perhaps because they had learned to adopt a more detached attitude regarding the outcomes of their interventions, and lowered their expectations concerning change (Farrenkopf, 1992).

### Intervention Strategies

To our knowledge, there have been no studies of the intervention strategies adopted by therapists who are still in contact with patients who have sexually reoffended during therapy. The most frequently reported reactions in this study were being sensitive to the experience of the patient, asking the patient what drove them to reoffend, and asking the patient why they had not been open with them. There were no significant gender-based differences in participants’ intervention strategies. Participants with fewer than 10 years of experience were more likely to be sensitive to the experience of the patient and to attempt to ensure that the patient realized their therapeutic progress despite the recidivism. This appears to correspond to Farrenkopf’s (1992) mission phase experienced by therapists in the first 5 years of practice with individuals who have committed sexual offenses. In this study, therapists with more than 10 years of experience were more likely to confront the patient about their lack of openness and failure to take advantage of the therapeutic work; this appears to correspond to Farrenkopf’s anger phase.

The results of this study suggest that participants’ reactions depended on the way they learned of their patient’s recidivism. Participants who were informed by the patient were more likely than those who were informed by a third party to exhibit empathy for the patient. The openness embodied by the patient’s self-reporting probably influenced the participants’ view of therapeutic alliance. One participant reported that she had been “happy that the patient had trusted her enough to admit his recidivism, which involved voyeurism. I believe that this was a sign that there was alliance after all.” Participants who learned of the recidivism from the patient were more likely to want to maintain the patient’s confidentiality regarding the recidivism and continue the therapeutic work. Participants who learned of the recidivism from a third party were more likely to be disappointed in their patient, who, they believed, had not shown sufficient openness; this tended to favor doubts about the strength of the therapeutic alliance. One participant reported “feeling like I was working harder than the patients.”

### Limitations

Although this study was innovative and helps fill a knowledge gap related to the experience of therapists working with individuals who have committed sexual offenses, it does have some limitations. First, the study is exploratory in nature, and its results should not be considered representative of every therapist working with individuals who have committed sexual offenses. In fact, our sample was rather small, and psychiatrists and psychologists in private practice were underrepresented. Furthermore, our sample included only therapists working in Quebec (Canada).

It is somewhat surprising that most of the therapists who participated in our study reported a case of sexual recidivism during treatment. This may be because only therapists who were comfortable disclosing recidivism chose to participate in the study, with therapists wishing to avoid reliving the unpleasant experience or with concerns regarding confidentiality declining to participate. As well, some therapists may have stopped working with this clientele following a case of sexual recidivism. However, a more likely reason for such a high rate is the fact that most of the participants had worked for many years in the field, and the cumulative probability of recidivism in these circumstances is high. However, to be more specific about the impact of years of experience, future research should also include questions on the number of clients seen.

Second, the responses provided on the questionnaire may not have been sufficiently comprehensive. For example, the questionnaire did not collect information on the intensity and duration of respondents’ emotions. Furthermore, respondents had to complete the questionnaire retrospectively, which means that the reported cognitions, emotions, and intervention strategies may not be accurate due to memory recollection. As well, participants who have had multiple patients sexually reoffend may have confused or merged cases. The use of “the case which affected you the most” helped clarify the research question and elicit memories that we expected to be the most vivid, but this strategy may have resulted in an over-representation of intense emotions and cognitions absent from less striking cases.

Third, the study design was primarily quantitative, with the qualitative component limited to optional comments, which were not often provided by participants. In order to further investigate this issue, it would be useful to conduct a fully qualitative study based on open-ended, semi-structured interviews. This would allow respondents to provide a dynamic narrative of the way they learned about the recidivism of their patients, their reactions to the recidivism, and their coping and intervention strategies. Furthermore, if done in a prospective design, diary methods could be integrated to better capture the duration and intensity of reactions following recidivism.

### Implications for Practice

Recidivism during treatment is part of the reality of therapists who see patients who have committed sexual offenses. In this study, 50 of the 59 respondents had a patient who sexually reoffended. Of these 50, 35 continued to see their patient after the recidivism. Participants’ cognitive and emotional reactions had an impact on their personal and professional lives, and may also have played a role in the intervention strategies put in place with the reoffending patient. The results of this study suggest that some emotional reactions, such as anxiety, may be associated with more empathy-oriented intervention strategies, while others, such as anger, may be associated with more accountability-oriented and guilt-driving ones. Consequently, it is essential that therapists be able to express their cognitions and emotions related to the sexual recidivism of a patient. Some will do so informally with those close to them or with work colleagues. Others will do so in the context of supervisory meetings or personal therapy. But in all cases, the therapist’s professional environment should favor the expression of these feelings, through debriefing sessions or meetings in which therapists attempt to understand the factors that could have led to their patient’s recidivism. It is equally important that the work environment be supportive, so that therapists are comfortable opening up without fear of being judged by their colleagues or employer. Moulden and Firestone (2010) reported that debriefing meetings and clinical supervision sensitize clinicians to the impact of working with individuals who have committed sexual offenses, normalize these impacts, and provide clinicians with support. Supervision is thus one of the most effective coping strategies (Ellerby et al., 1993; Ennis & Horne, 2003; Moulden & Firestone, 2010). Co-therapy and co-supervision can also help.

In light of these reports and of our results, a number of specific recommendations concerning strategies for coping with patient recidivism during treatment can be formulated. First, pairing less-experienced therapists with more-experienced ones may help the former put the recidivism of their patient into perspective, as our results indicate that less-experienced therapists are more likely to question their efficacy and experience more disillusionment than more-experienced ones. Conversely, this pairing may also lead more-experienced therapists to have a more positive attitude towards recidivists, as less-experienced therapists in this study displayed more empathy towards their patients and were more inclined to recognize progress made in treatment. Second, co-therapy and co-supervision may help both female and male therapists develop a more balanced view of their situation, leading to decreased disappointment (internal attribution of responsibility) by female therapists and decreased anger at, and blame of, the patient (external attribution of responsibility) by male therapists. Co-therapy and co-supervision may also help therapists react in a more nuanced way to news of a patient’s recidivism, whether this news comes from patients or others. For example, the implementation of a mentoring model, through a community of practice where therapists can discuss and explore recidivism in treatment (and other issues), could be worthwhile to prevent professional burnout and strengthen self-confidence.

Therapists should receive more information about the probability of recidivism during treatment, their possible reactions to recidivism, and the best way to manage their reactions. Some studies have suggested that erosion and burnout can be avoided by limiting the number of hours of therapeutic work with individuals who have committed sexual offenses, and diversifying both tasks and clienteles (Edmunds, 1997; Scheela, 2001). Self-care strategies may also prove useful. Moulden and Firestone (2010) indicate that the first step in self-care is recognizing the potential personal impact of work with individuals who have committed sexual offenses. They suggest that clinicians reflect on their personal experiences, biases, and reactions, especially by reviewing their thoughts, emotions, and intervention strategies. Practicing sports or other physical activities, developing diversified interests unrelated to work, finding a balance between work and leisure, undertaking personal therapy, and avoiding media reports related to sexual offenses are other self-care strategies that could be relevant (Ellerby, 1998; Scheela, 2001).

## References

[bibr1-10790632231153636] Brady J. L. Guy J. D. Poelstra P. L. Brokaw B. F. (1999). Vicarious traumatization, spirituality, and the treatment of sexual abuse survivors: A national survey of women psychotherapists. Professional Psychology: Research and Practice, 30(4), 386–393. 10.1037/0735-7028.30.4.386

[bibr2-10790632231153636] Brouillette-Alarie S. (2021). Quelles sont les interventions les plus efficaces pour réduire le risque de récidive des auteurs de crimes sexuels? What are the most effective interventions for reducing the risk of recidivism by individuals having committed sexual crimes. Revue Internationale de Criminologie et de Police Technique et Scientifique, 74, 3–22.

[bibr3-10790632231153636] Chrestman K. R. (1999). Secondary exposure to trauma and self reported distress among therapists. In Stamm B. H. (Ed.), Secondary traumatic stress: Self care issues for clinicians, researchers and educators (2nd ed., pp. 37–47). Sidran Institute.

[bibr4-10790632231153636] Chudzik L. Ashchieri F. (2013). Clinical relationships with forensic clients: A three-dimensional model. Aggression and Violent Behavior, 18(6), 722–731. 10.1016/j.avb.2013.07.027

[bibr5-10790632231153636] Collins J. M. (2003). Impact of patient suicide on clinicians. Journal of the American Psychiatric Nurses Association, 9(5), 159–162. 10.1016/S1078-3903(03)00221-0

[bibr6-10790632231153636] Dean C. Barnett G. (2011). The personal impact of delivering a one-to-one treatment programme with high-risk sexual offenders: Therapists’ experiences. Journal of Sexual Aggression, 17(3), 304–319. 10.1080/13552600.2010.506577

[bibr7-10790632231153636] Edmunds S. B. (1997). Personal impact of working with sex offenders. In Edmunds S. B. (Ed.), Impact: Working with sexual abusers (pp. 11–29). Safer Society Press.

[bibr8-10790632231153636] Ellerby L. (1997). Impact on clinicians: Stressors and providers of sex-offender treatment. In Edmunds S. B. (Ed), Impact: Working with sexual abusers (pp. 51–60). Safer Society Press.

[bibr9-10790632231153636] Ellerby L. (1998). Providing clinical service to sex offenders: Burnout, compassion fatigue and moderating variables. National Library of Canada Publication No. . 0-612-31976-8 (Doctoral dissertation, University of Manitoba) https://www.collectionscanada.gc.ca/obj/s4/f2/dsk2/tape15/PQDD_0027/NQ31976.pdf

[bibr10-10790632231153636] Ellerby L. Gutkin B. Smith T. Atkinson R. (1993). Treating sex offenders: The impact on clinicians. Annual conference of the association for the treatment of sexual abusers. Paper presented at the 12th.

[bibr11-10790632231153636] Ennis L. Horne S. (2003). Predicting psychological distress in sex offender therapists. Sexual Abuse: A Journal of Research and Treatment, 15(2), 149–157. 10.1177/107906320301500205 12731149

[bibr12-10790632231153636] Farber B. A. Heifetz L. J. (1982). The process and dimensions of burnout in psychotherapists. Professional Psychology, 13(2), 293–301. 10.1037/0735-7028.13.2.293

[bibr13-10790632231153636] Farrenkopf T. (1992). What happens to therapists who work with sex offenders? Journal of Offender Rehabilitation, 18(3-4), 217–224. 10.1300/J076v18n03_16

[bibr14-10790632231153636] Figley C. R. (1995). Compassion fatigue: Coping with secondary traumatic stress disorder in those who treat the traumatized. Routledge.

[bibr15-10790632231153636] Freeman-Longo R. E. (1997). Introduction: A personal and professional perspective on burnout. In Edmunds S. B. (Ed.), Impact: Working with sexual abusers (pp. 5–9). Safer Society Press.

[bibr16-10790632231153636] Gannon T. A. Olver M. E. Mallion J. S. James M. (2019). Does specialized psychological treatment for offending reduce recidivism? A meta-analysis examining staff and program variables as predictors of treatment effectiveness. Clinical Psychology Review, 73, 101752. 10.1016/j.cpr.2019.101752 31476514

[bibr17-10790632231153636] Grad O. T. Zavasnik A. Groleger U. (1997). Suicide of a patient: Gender differences in bereavement reactions of therapists. Suicide and Life-Threatening Behavior, 27(4), 379–386. 10.1111/j.1943-278X.1997.tb00517.x 9444733

[bibr18-10790632231153636] Hanson R. K. Bourgon G. Helmus L. Hodgson S. (2009). The principle of effective correctional treatment also applies to sexual offenders: A meta-analysis. Criminal Justice and Behavior, 36(9), 865–891. 10.1177/0093854809338545

[bibr19-10790632231153636] Hardeberg Bach M. Demuth C. (2018). Therapists’ experiences in their work with sex offenders and people with pedophilia: A literature review. Europe’s Journal of Psychology, 14(2), 498–514. 10.5964/ejop.v14i2.1493 PMC6016037 30008959

[bibr20-10790632231153636] Hardeberg Bach M. Demuth C. (2019). Therapists’ personal experiences in their work with clients who have sexually offended against children: A phenomenological study. Journal of Child Sexual Abuse, 28(7), 799–818. 10.1080/10538712.2019.1592273 30907705

[bibr21-10790632231153636] Hendin H. Lipschitz A. Maltsberger J. T. Haas A. P. Wynecoop S. (2000). Therapists’ reactions to patients’ suicides. The American Journal of Psychiatry, 157(12), 2022–2027. 10.1176/appi.ajp.157.12.2022 11097970

[bibr22-10790632231153636] Jackson K. E. Holzman C. Barnard T. Paradis C. (1997). Working with sex offenders: The impact on practitioners. In Edmunds S. B. (Ed), Impact: Working with sexual abusers (pp. 61–73). Safer Society Press.

[bibr23-10790632231153636] Lea S. Auburn T. Kibblewhite K. (1999). Working with sex offenders: The perceptions and experiences of professionals and paraprofessionals. International Journal of Offender Therapy and Comparative Criminology, 43(1), 103–119. 10.1177/0306624X99431010

[bibr24-10790632231153636] McCann I. L. Pearlman L. A. (1990). Vicarious traumatization: A framework for understanding the psychological effects of working with victims. Journal of Traumatic Stress, 3(1), 131–149. 10.1007/BF00975140

[bibr25-10790632231153636] Moulden H. M. Firestone P. (2007). Vicarious traumatization: The impact on therapists who work with sexual offenders. Trauma, Violence, and Abuse, 8(1), 67–83. 10.1177/152838006297729 17204600

[bibr26-10790632231153636] Moulden H. M. Firestone P. (2010). Therapist awareness and responsibility in working with sexual offenders. Sexual Abuse, 22(4), 374–386. 10.1177/1079063210382047 20947698

[bibr27-10790632231153636] Pearlman L. A. Mac Ian P. S. (1995). Vicarious traumatization: An empirical study of the effects of trauma work on trauma therapists. Professional Psychology: Research and Practice, 26(6), 558–565. 10.1037/0735-7028.26.6.558

[bibr28-10790632231153636] Rice M. E. Harris G. T. (2005). Comparing effect sizes in follow-up studies: ROC area, cohen’s d, and r. Law and Human Behavior, 29(5), 615–620. 10.1007/s10979-005-6832-7 16254746

[bibr29-10790632231153636] Rich K. D. (1994). Outpatient group therapy with adult male sex offenders: Clinical issues and concerns. Journal for Specialists in Group Work, 9(2), 120–128. 10.1080/0193392908413771

[bibr30-10790632231153636] Scheela R. A. (2001). Sex offender treatment: Therapists’ experiences and perceptions. Issues in Mental Health Nursing, 22(8), 749–767. 10.1080/01612840152713009 11881178

[bibr31-10790632231153636] Schmucker M. Lösel F. (2015). The effects of sexual offender treatment on recidivism: An international meta-analysis of sound quality evaluations. Journal of Experimental Criminology, 11(4), 597–630. 10.1007/s11292-015-9241-z

[bibr33-10790632231153636] Shelby R. A. Stoddart R. M. Taylor K. L. (2001). Factors contributing to levels of burnout among sex offender treatment providers. Journal of Interpersonal violence, 16(1), 1205–1217. 10.1177/088626001016011006

[bibr34-10790632231153636] Slater C. Lambie I. (2011). The highs and lows of working with sexual offenders: A New Zealand perspective. Journal of Sexual Aggression, 17(3), 320–334. 10.1080/13552600.2010.519056

[bibr35-10790632231153636] Steed L. Bicknell J. (2001). Trauma and the therapist: The experience of therapists working with the perpetrators of sexual abuse. The Australian Journal of Disaster and Trauma Studies, 1, 30–37.

[bibr36-10790632231153636] Valente S. M. Sanders J. M. (2002). Nurses’ grief reactions to a patient’s suicide. Perspectives in Psychiatric Care, 38(1), 5–14. 10.1111/j.1744-6163.2002.tb00650.x 11939086

[bibr37-10790632231153636] Ward T. (2010). Is offender rehabilitation a form of punishment? The British Journal of Forensic Practice, 12(4), 4–13. 10.5042/bjfp.2010.0610

[bibr38-10790632231153636] Way I. Van Deusen K. M. Martin G. Applegate B. Jandle D. (2004). Vicarious trauma: A comparison of clinicians who treat survivors of sexual abuse and sexual offenders. Journal of Interpersonal Violence, 19(1), 49–71. 10.1177/0886260503259050 14680529

[bibr39-10790632231153636] Woodhouse A. Craven-Staines S. (2021). A systematic review exploring the role of gender in staffs experience of working with sexual offenders. Journal of Criminological Research, Policy and Practice, 7(4), 373–383. 10.1108/JCRPP-04-2021-0018

